# Associations between women’s childhood maltreatment and thyroid function before and during pregnancy

**DOI:** 10.1038/s41598-026-48820-9

**Published:** 2026-04-25

**Authors:** Chirag M. Vyas, Bohao Wu, Shruthi Mahalingaiah, Rajarshi Mukherjee, Natalie Slopen, Marc G. Weisskopf, Jorge E. Chavarro, Andrea L. Roberts

**Affiliations:** 1 Department of Psychiatry, Massachusetts General Hospital, Boston, MPH, 125 Nashua Street, #7102A, Boston, MA 02114 USA; 2 Department of Environmental Health, Harvard T.H. Chan School of Public Health, Boston, MA USA; 3 Department of Epidemiology, Columbia University Mailman School of Public Health, New York, NY USA; 4 Department of Social and Behavioral Sciences, Harvard T.H. Chan School of Public Health, Boston, MA USA; 5 Department of Nutrition, Harvard T.H. Chan School of Public Health, Boston, MA USA; 6 Department of Epidemiology, Harvard T.H. Chan School of Public Health, Boston, MA USA; 7 Channing Division of Network Medicine, Department of Medicine, Harvard Medical School, Brigham and Women’s Hospital, Boston, MA USA

**Keywords:** Childhood maltreatment, Thyroid function, Pregnancy, Abuse, Neglect, Hormones, Endocrinology, Trauma, Translational research, Epidemiology

## Abstract

Maternal childhood maltreatment has been associated with complications during pregnancy and neurodevelopmental disorders in offspring. Thyroid dysfunction could be a mechanistic pathway underlying this relationship. The thyroid is vital for reproductive health in women, maintaining menstrual cycles, fertility, and pregnancy. However, little is known how childhood maltreatment affects thyroid function in women contemplating pregnancy and those who are pregnant. Participants were from the Nurses’ Health Study 3, including 217 women contemplating pregnancy and 156 pregnant women, all reporting childhood maltreatment using a validated 28-item Childhood Trauma Questionnaire. Thyroid function tests were plasma thyroid-stimulating hormone (TSH), free triiodothyronine (FT3), free thyroxine (FT4), and anti-thyroperoxidase antibody (TPOAb). Regression analyses were conducted. Among women contemplating pregnancy (n = 217), childhood maltreatment was consistently associated with lower FT3 levels, but not with FT4, TSH, or TPOAb levels. FT3 levels were 5% to 7% lower in those with mild to severe childhood maltreatment compared to those without. Among pregnant women (n = 156), no significant associations were observed between childhood maltreatment and thyroid function. In conclusion, childhood maltreatment was associated with low thyroid function (e.g., FT3) among women contemplating pregnancy, but not among pregnant women. Future large, prospective, racially and ethnically diverse studies are needed to confirm our findings.

**Supplementary Information:**

The online version contains supplementary material available at 10.1038/s41598-026-48820-9.

## Introduction

The thyroid gland plays a vital role for women of childbearing age, influencing various aspects of reproductive health such as menstrual regularity, conception, and optimal fetal neurodevelopment during pregnancy^1–3^. External stressors such as childhood maltreatment can disrupt thyroid function, potentially linking maternal early-life adversity to worse health outcomes in offspring^4–7^. Maternal childhood maltreatment—defined as neglect or physical, emotional, or sexual abuse experienced by the mother during her own childhood—has been associated with an increased risk of neurodevelopmental disorders in offspring, including autism spectrum disorder (ASD) and attention-deficit/hyperactivity disorder (ADHD)^8–10^. Despite its potential importance, research on how childhood maltreatment affects thyroid function in women of childbearing age is limited.

Evidence indicates that childhood maltreatment affects thyroid function during adulthood via changes to hypothalamic-pituitary-thyroid (HPT)- and hypothalamic–pituitary–adrenal (HPA)-axis functioning. Childhood maltreatment can disrupt the HPT axis through stable epigenetic modifications that alter gene expression involved in stress response and endocrine regulation, potentially leading to long-term changes in thyroid function^5,11^. Animal studies have demonstrated that early-life stress induces long-lasting epigenetic alterations in genes regulating the HPT axis, such as altered DNA methylation patterns in hypothalamus and thyroid tissue. These epigenetic alterations are associated with dysregulated thyroid hormone levels, suggesting a mechanistic link between early-life adversity and thyroid dysfunction^12,13^. Additionally, chronic stress from maltreatment can cause prolonged increases in cortisol and disrupt the HPA axis, which interacts with the HPT axis^14^. Because the HPA and HPT axes interact via shared hypothalamic pathways, cortisol imbalances may inhibit thyroid hormone production and alter thyroid regulation.

Previous studies have examined the relationship between childhood maltreatment and thyroid function in non-pregnant individuals, including adolescents and those with psychiatric disorders^15–18^ and have reported thyroid dysfunction in those exposed versus unexposed to early life trauma. In addition, some evidence indicates that thyroid dysfunction prior to pregnancy, regardless of thyroid levels during pregnancy, is associated with ASD and ADHD in offspring^6,7^. However, research including women of childbearing age for investigating the relationship between childhood maltreatment and thyroid function before and during pregnancy is sparse^5^. Investigating this relationship across different reproductive stages—whether in women contemplating pregnancy or who are pregnant—could uncover endocrine-related mechanisms that may account for the association of maternal childhood maltreatment with adverse offspring health. These insights may lead to better preventive strategies, ultimately reducing risk of adverse neurodevelopmental outcomes in children.

In this study, we examined the relationship between childhood maltreatment and thyroid function in two groups: women contemplating pregnancy and pregnant women. We hypothesized that the experience of childhood maltreatment, in the form of emotional abuse, physical abuse, sexual abuse, emotional neglect, or physical neglect, would be associated with altered levels of thyroid function before and during pregnancy.

## Methods

### Study sample

Participants were members of the Nurses’ Health Study 3 (NHS3), an ongoing, prospective, web-based cohort study^19^. NHS3 was launched in 2010 and includes both female and male registered nurses and nursing students in the US and Canada who were born on or after January 1, 1965. By January 2023, 50,150 individuals had consented to participate, and 36,010 had completed at least one follow-up questionnaire. Since enrollment, participants have received follow-up questionnaires approximately every six months to provide updates on their lifestyle, reproductive, and medical information.

**Fig. 1:**
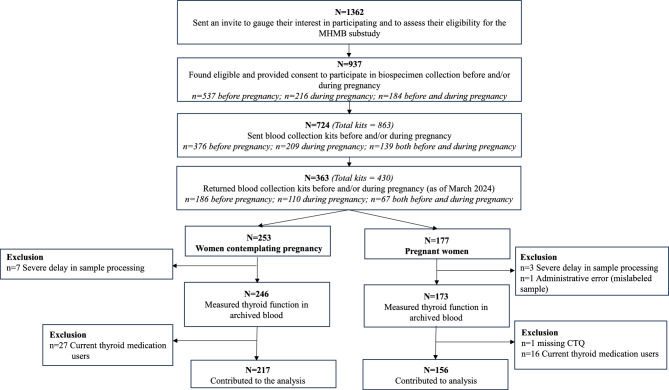
Flow chart of study participation.

In October 2020, the Maternal Health and Maternal Biology (MHMB) sub-study was established within NHS3 to investigate the associations between childhood maltreatment and biomarkers before and during pregnancy. Each NHS3 questionnaire asked women whether they planned to become pregnant in the next year or if they were currently pregnant. Based on their responses, 1362 women—either contemplating pregnancy or currently pregnant with a singleton under 24 weeks of gestation— and who were currently living in the continental U.S. were invited to participate in the MHMB sub-study (Fig. 1). Of these, 937 women were eligible and consented to provide biospecimens before and/or during pregnancy: 537 consented to provide samples while contemplating pregnancy, 216 during pregnancy, and 184 for both time periods. A total of 724 biospecimen kits were sent: 376 kits to women contemplating pregnancy, 209 kits during pregnancy, and 139 for both periods. As of March 2024, a total of 363 kits have been returned and processed: 253 from women contemplating pregnancy and 177 from women during pregnancy.

This study adhered to ethical guidelines and all procedures involving human participants were conducted in accordance with the Declaration of Helsinki. Informed consent was obtained from all participants enrolled in the sub-study. Study protocols were approved by the Institutional Review Board of Mass General Brigham.

#### Biospecimen collection and handling procedures

Briefly, women contemplating pregnancy were sent a biospecimen collection kit immediately upon consenting (typically within one week). Women who were pregnant received their kit no earlier than week 14 of pregnancy. Participants had blood drawn at a nearby Quest Diagnostics location or through an at-home phlebotomy service. Refrigerated blood samples were then shipped to the Channing Laboratory of Mass General Brigham. Upon receipt, samples were centrifuged, aliquoted, and frozen at − 80 °C. Frozen samples were later sent in a single shipment to the Rifai Laboratory of Boston Children’s Hospital for thyroid function assays.

### Ascertainment of childhood maltreatment

Women who consented to participate in the MHMB sub-study were asked to complete a questionnaire in which childhood maltreatment was assessed using the short version of the Childhood Trauma Questionnaire (CTQ)^20,21^. The CTQ consists of 28 items, with statements beginning with the phrase “When I was growing up, during my 18 years of life…” Out of 28 CTQ items, 25 measure childhood maltreatment, including 5 abuse and neglect subscales (physical abuse, emotional abuse, sexual abuse, physical neglect, and emotional neglect), each consisting of 5 items respectively. The remaining 3 CTQ items are designed to measure Minimization/Denial (M/D). Since the validity of these items have not been established in the literature, we did not include them in the computation of the CTQ score^22^.

The response options for all 5 abuse and neglect subscales range from “never true” (1 point) to “very often true” (5 points). Responses to positive items—such as whether the respondent felt loved, whether they were made to feel important, and whether they were looked out for—were reverse coded so that “never true” corresponds to 5 points and “very often true” corresponds to 1 point. Thus, each item can range from 1 to 5 points, and each subscale can range from 5 to 25 points. We computed a total CTQ score (range: 25–125 points) by summing responses to all questions. Leveraging prior work^21^, we categorize a total CTQ score into four categories to reflect the severity of childhood maltreatment: none (25 points), minimal (26–29 points), mild (30–36 points), and moderate or severe (> 36 points).

### Thyroid function

In both groups—women contemplating pregnancy and pregnant women—we conducted four thyroid function tests: thyroid-stimulating hormone (TSH), free triiodothyronine (FT3), free thyroxine (FT4), and anti-thyroperoxidase antibody (TPOAb). In both groups, samples were run in two batches with identical laboratory and handling procedures, and the mean values of thyroid function tests were comparable between two batches, minimizing the potential effects of batch variation. Laboratory personnel were blinded to maltreatment and pregnancy status. The plasma concentrations of TSH, FT3, FT4, and TPOAb were measured using the Food and Drug Administration-approved electrochemiluminescence immunoassay for clinical use (*Roche Cobas 6000 system, Roche Diagnostics, Indianapolis, IN*). The lower limits of detection (LOD) were 0.005 µIU/mL for TSH, 0.6 pmol/L for FT3, 0.05 ng/dL for FT4, and 5.0 IU/mL for TPOAb. All participants had measured values for FT3 and FT4. TSH values were below the LOD for 19 out of 219 women contemplating pregnancy and for 20 out of 156 pregnant women. Additionally, TPOAb were below the LOD in majority of participants (≥ 80%) in both groups. For participants with TSH and TPOAb values below the LOD, we imputed these as the corresponding LLOD for each thyroid function test divided by the square root of 2^23^.

To minimize the impact of extreme outliers, we winsorized thyroid function test values at the bottom and top 1.0% and then inspected the distributions for normality. Additionally, as FT3, FT4, and TSH were right skewed, they were log-transformed to improve normality. TPOAb was categorized as a binary variable: positive (> 9 IU/mL) or negative (≤ 9 IU/mL)^24^. Coefficients of variation (CV) were calculated from blinded duplicate aliquots of 42 participant samples, assessed in two batches. The range of mean CVs across two batches was: TSH = 1.1%-3.1%, FT3 = 0.7%-2.1%, FT4 = 0.8%-2.7%, and TPOAb = 0.9%-7.7%. Since each of these 42 samples was analyzed twice, we used the average of the two measurements for each sample in our analysis.

### Assessment of covariates, potential mediators, and exclusion variables

Participants reported their date of birth, race (White, Black, Asian, Native Hawaiian, or other Pacific Islander, American Indian or Alaska Native, and other ancestry) and height on the NHS3 baseline questionnaire. A supplemental questionnaire administered during the blood draw collected additional information, including hours since the last food or beverage consumption (excluding water), time of morning awakening (“when your eyes first opened”), time of blood draw, use of thyroid medications in the past 48 h (for example, Levothyroxine, Synthroid, Levoxyl, Unithroid, Tirosint), gestational age, weight, smoking status, and marijuana use. Additionally, we queried educational attainment, history of cancer and history of autoimmune disorder from NHS3 follow-up questionnaires. Hours since waking were calculated by subtracting the time of morning awakening ("when your eyes first opened") from the time of the blood draw.

Covariates were selected based on theoretical considerations and known associations with childhood maltreatment, thyroid function, or both^3,25,26^. These covariates included age (in years), self-reported race (White versus other), pregnancy trimesters using gestational age, hours since waking, and hours since the most recent food or drink consumption. Additionally, we considered potential mediators that might be on the path between childhood maltreatment and thyroid function, such as educational attainment, body mass index (BMI), smoking use, marijuana usage, history of cancer and history of autoimmune disorders. Data on past 48-h thyroid medication use was used to exclude individuals from analysis.

### Final sample for thyroid function analysis

As of March 2024, 253 participants returned their blood kits while contemplating pregnancy (see Fig. 1). After excluding 7 participants due to shipping delays that resulted in poor-quality samples, we measured thyroid function in 246 participants, all of whom had data on childhood maltreatment. Following the exclusion of 27 participants who were current users of thyroid medication, 217 participants contributed to the analysis of childhood maltreatment and thyroid function in this group.

As of March 2024, 177 pregnant women returned their blood kits. After excluding 4 participants due to shipping delays resulting in poor-quality samples, we measured thyroid function among 173 participants. In this group, after further excluding 1 participant with missing CTQ data and 16 current users of thyroid medication, 156 participants contributed to the analysis of childhood maltreatment and thyroid function in pregnant women.

### Statistical analyses

We conducted separate analyses for two groups: women contemplating pregnancy and pregnant women.

#### Women contemplating pregnancy

Participants’ characteristics, including sociodemographic, lifestyle, and health-related factors at baseline, were stratified by categories of childhood maltreatment: none (25 points), minimal (26–29 points), mild (30–36 points), and moderate or severe (> 36 points). We also calculated Spearman rank correlations between thyroid function tests and between age and thyroid function tests.

To examine the association between childhood maltreatment and TSH, FT3, and FT4, we fit linear regression models and reported percent differences with 95% confidence intervals (CIs). Normality of residuals was assessed using the Shapiro–Wilk test and visual inspection of Q-Q plots, and homogeneity of variance was evaluated using Levene’s test. Each log-transformed thyroid function test level was modeled as the dependent variable, with CTQ score (a continuous variable to assess trends) and CTQ categories (minimal, mild, or moderate and severe versus none) as independent variables. In the first model, we adjusted for age, self-reported race, hours since waking, and hours since the most recent food or drink consumption. In the second model, we additionally adjusted for potential mediators, such as educational attainment, BMI, smoking, marijuana usage, history of cancer and history of autoimmune disorders. We used multivariable logistic models to examine the relationship between childhood maltreatment and the likelihood of positive TPOAb. Multicollinearity was assessed using variance inflation factors, and linearity in the logit was evaluated for continuous predictors. Separate models were also fit further adjusted for potential covariates and mediators, and odds ratios (ORs) with 95% CIs were presented. Secondarily, we examined associations between the five abuse and neglect sub-scales of the CTQ (physical abuse, emotional abuse, sexual abuse, physical neglect, and emotional neglect) and thyroid function tests.

#### Pregnant women

We compared participants’ characteristics according to severity of childhood maltreatment. We computed Spearman-rank correlations between thyroid function tests and further stratified by pregnancy trimester. We used the same modeling and covariate strategies as with the group of women contemplating pregnancy and included gestational age in the two models: linear regression models to examine the relationship between childhood maltreatment and FT3, FT4, and TSH, and logistic regression models to examine the relationship between childhood maltreatment and likelihood of positive TPOAb. Secondarily, we examined associations between the five abuse and neglect sub-scales of the CTQ (physical abuse, emotional abuse, sexual abuse, physical neglect, and emotional neglect) and thyroid function tests.

All analyses were conducted using SAS version 9.4 (SAS Institute). All statistical tests were two-sided, and a significance level of α = 0.05 was used.

## Results

### Women contemplating pregnancy

#### Baseline characteristics

Among 219 women contemplating pregnancy, the mean (standard deviation [SD]) age was 34.7 (4.1) years, and 89.0% were White. Among these women, 55 (25.1%) experienced moderate or severe maltreatment during childhood. Participants’ characteristics by severity of childhood maltreatment are shown in Table 1. Compared to those who did not experience childhood maltreatment, individuals reporting moderate or severe childhood maltreatment were slightly older and higher BMI, and more likely to be non-White, to have ever smoked or used marijuana, and to have a history of cancer. FT3 and FT4 levels were significantly positively correlated with each other (Spearman-rank correlation coefficient, ρ = 0.39, *p* =  < 0.0001; Table S1), while TSH levels were negatively correlated with FT4 (ρ = − 0.13, *p* = 0.05; Table S1). Older age was significantly associated with lower FT3 levels (Spearman rank correlation coefficient, ρ = − 0.20, *p* = 0.003; Table S2). Thyroid function test values did not differ by level of childhood maltreatment, except that FT3 levels were slightly lower in those who reported moderate or severe childhood maltreatment compared to those with no childhood maltreatment.

**Table 1:** Characteristics of women contemplating pregnancy with data on thyroid function in the sample and by severity levels of childhood maltreatment.

Characteristics	Sample (n = 219)	Severity levels of childhood maltreatment			
		None (CTQ score: 25 points) (n = 29)	Minimal (CTQ score: 26–29 points) (n = 69)	Mild (CTQ score: 30–36 points) (n = 66)	Moderate or severe (CTQ score: > 36 points) (n = 55)
Age at MHMB sub-study enrollment, Mean (SD), yrs	34.7 (4.1)	34.6 (3.9)	34.4 (4.3)	34.5 (3.7)	35.5 (4.6)
Self-reported race, n (%)					
White	195 (89.0)	28 (96.6)	63 (91.3)	58 (87.9)	46 (83.6)
Other	24 (11.0)	1 (3.5)	6 (8.7)	8 (12.1)	9 (16.4)
Education, n (%)					
Bachelor or lower	92 (42.0)	13 (44.8)	38 (55.1)	17 (25.8)	24 (43.6)
Master’s or Doctorate	127 (58.0)	16 (55.2)	31 (44.9)	49 (74.2)	31 (56.4)
BMI, kg/m^2^, Median (IQR)	24.6 (22.0 to 28.8)	23.2 (22.1 to 26.6)	24.0 (21.6 to 29.3)	25.4 (22.2 to 30.2)	25.3 (22.3 to 28.4)
Ever smoker, n (%)	30 (13.7)	2 (6.9)	8 (11.6)	8 (12.1)	12 (21.8)
Ever consumed marijuana, n (%)	129 (58.9)	14 (48.3)	36 (52.2)	42 (63.6)	37 (67.3)
History of cancer, n (%)	11 (5.0)	1 (3.5)	3 (4.4)	3 (4.6)	4 (7.3)
History of autoimmune disorders, n (%)	14 (6.4)	0 (0.0)	5 (7.3)	7 (10.6)	2 (3.6)
Free T3, pmol/L, Median (IQR)	4.4 (4.0 to 4.7)	4.6 (4.2 to 5.1)	4.4 (4.0 to 4.7)	4.2 (3.9 to 4.7)	4.3 (4.0 to 4.6)
Free T4, ng/dL, Median (IQR)	1.1 (1.1 to 1.2)	1.2 (1.1 to 1.3)	1.1 (1.1 to 1.3)	1.1 (1.0 to 1.2)	1.1 (1.1 to 1.2)
TSH, uIU/mL, Median (IQR)	1.6 (1.2 to 2.2)	1.6 (1.4 to 2.6)	1.6 (1.2 to 2.1)	1.7 (1.3 to 2.4)	1.8 (1.1 to 2.2)
Positive TPOAb, n (%)					
Yes (> 9 IU/mL)	20 (9.1)	3 (10.3)	6 (8.7)	7 (10.6)	4 (7.3)
No (≤ 9 IU/mL)	199 (90.9)	26 (89.7)	63 (91.3)	59 (89.4)	51 (92.7)
Blood draw since wake up (in hours), Median (IQR)	3.7 (2.3 to 5.3)	3.3 (2.1 to 4.8)	4.2 (2.7 to 5.9)	3.8 (2.3 to 5.6)	3.3 (2.2 to 5.0)
Blood draw since last eat or drink (in hours), Median (IQR)	2.0 (1.0 to 4.0)	1.5 (1.0 to 9.0)	1.0 (1.0 to 3.0)	2.0 (1.0 to 8.0)	2.0 (1.0 to 9.5)

*MHMB* Maternal Health and Maternal Biology, *CTQ* childhood trauma questionnaire, *BMI* body mass index, *TSH* thyroid stimulating hormone, *IU* international unit, *TPO* thyroid peroxidase, *SD* standard deviation, *IQR* interquartile range.

#### Associations between childhood maltreatment and thyroid function tests

Among women contemplating pregnancy, those who reported childhood maltreatment had lower FT3 levels than those who did not, after adjusting for age, self-reported race, hours since awakening, and time since last eating or drinking. Specifically, women with minimal, mild, and moderate or severe childhood maltreatment had 5%, 7% and 5% lower FT3 levels, respectively (Table 2, Model 1). However, there was no consistent linear trend across maltreatment severity (p-trend: 0.39). Results remained similar in a model further adjusted for potential mediators (Table 2, Model 2). No consistent associations were observed between childhood maltreatment and FT4 or TSH levels (Table 2). Specifically, women with mild childhood maltreatment had approximately 7% lower FT4 levels compared to those who did not report childhood maltreatment (percent difference [95% CI]: − 6.8% [− 11.6% to − 1.7%]), but no such association was observed among those who reported moderate or severe maltreatment versus no maltreatment. Percent differences in TSH levels across childhood maltreatment severity were not statistically significant, and confidence intervals were wide. Childhood maltreatment was not significantly associated with positive TPOAb among women contemplating pregnancy (Table 3). We did not find significant associations between the abuse and neglect sub-scales of the CTQ and thyroid function tests in this sample (Table S3).

**Table 2:** Percent difference (95% CI) in free T3, free T4 and TSH levels among women contemplating pregnancy, according to severity levels of childhood maltreatment (n = 219).

Severity levels of childhood maltreatment	Free T3, pmol/L		Free T4, ng/dL		TSH, uIU/mL	
	Percent difference (95% CI)	p-trend^c^	Percent difference (95% CI)	p-trend^c^	Percent difference (95% CI)	p-trend^c^
Model 1^a^						
No (n = 29)	0 (Ref)	0.39	0 (Ref)	0.45	0 (Ref)	0.89
Minimal (n = 69)	− 4.5% (− 9.0% to 0.2%)		− 3.6% (− 8.5% to 1.7%)		− 14.4% (− 29.1% to 3.3%)	
Mild (n = 66)	− 6.9% (− 11.3% to − 2.3%)		− 6.8% (− 11.6% to − 1.7%)		− 3.6% (− 20.2% to 16.5%)	
Moderate or severe (n = 55)	− 5.3% (− 9.9% to − 0.5%)		− 1.7% (− 6.9% to 3.9%)		− 12.5% (− 28.0% to 6.3%)	
Model 2^b^						
No (n = 29)	0 (Ref)	0.40	0 (Ref)	0.44	0 (Ref)	0.98
Minimal (n = 69)	− 4.5% (− 9.1% to 0.3%)		− 3.1% (− 8.1% to 2.2%)		− 16.3% (− 30.9% to 1.5%)	
Mild (n = 66)	− 7.1% (− 11.6% to − 2.3%)		− 6.6% (− 11.5% to − 1.3%)		− 4.5% (− 21.6% to 16.4%)	
Moderate or severe (n = 55)	− 5.3% (− 10.0% to − 0.4%)		− 1.0% (− 6.4% to 4.6%)		− 12.6% (− 28.4% to 6.7%)	

*MHMB* Maternal Health and Maternal Biology, *CI* confidence interval.

^a^ Model 1 was adjusted for age at MHMB sub-study enrollment, race, blood draw since wake up (in hours), and blood draw since last eat or drink (in hours).

^b^ Model 2 was additionally adjusted for educational attainment, body mass index, ever smoked, ever used marijuana, history of cancer and history of autoimmune disorder.

^c^ p-trend was calculated by fitting models with severity of childhood maltreatment as a continuous variable.

**Table 3:** Association between severity levels of childhood maltreatment and odds of positive TPOAb among women contemplating pregnancy (n = 219).

Severity levels of childhood maltreatment	High vs. normal TPOAb (> 9 vs. ≤ 9 IU/mL)		
	Event rate, n (%)	Odds ratio (95% CI)	p-trend^c^
Model 1^a^			0.61
No	3/29 (10.3)	1.00 (Ref)	
Minimal	6/69 (8.7)	0.78 (0.18 to 3.43)	
Mild	7/66 (10.6)	0.98 (0.23 to 4.20)	
Moderate or severe	4/55 (7.3)	0.78 (0.16 to 3.84)	
Model 2^b^			0.71
No	3/29 (10.3)	1.00 (Ref)	
Minimal	6/69 (8.7)	0.55 (0.12 to 2.63)	
Mild	7/66 (10.6)	0.74 (0.15 to 3.60)	
Moderate or severe	4/55 (7.3)	0.66 (0.13 to 3.45)	

*MHMB* Maternal Health and Maternal Biology, *CI* confidence interval, *IU* international unit.

^a^ Model 1 was adjusted for age at MHMB study enrollment, race, blood draw since wake up (in hours), and blood draw since last eat or drink (in hours).

^b^ Model 2 was additionally adjusted for educational attainment, body mass index, ever smoked, ever used marijuana, history of cancer, and history of autoimmune disorder.

^c^ p-trend was calculated by fitting models with severity of childhood maltreatment as a continuous CTQ variable.

### Pregnant women

#### Baseline characteristics

Among 156 pregnant participants, the mean (SD) age was 34.4 (3.2) years, 93.0% were White, and the median (interquartile range) gestational age was 15.6 (12.0–23.0) weeks. Among pregnant participants, 38 (24.4%) experienced moderate or severe childhood maltreatment. Participants’ characteristics by severity of childhood maltreatment are shown in Table 4. Compared to those who did not experience childhood maltreatment, individuals reporting moderate or severe childhood maltreatment were slightly older, had lower educational attainment (bachelor’s degree or less), had higher BMI, and were more likely to be non-White, have ever smoked, and less likely to have an autoimmune disorder. Thyroid function tests did not differ by severity of childhood maltreatment. FT3 and FT4 levels were modestly correlated (ρ = 0.50, *p* < 0.0001; Table S4), while TSH levels were not correlated with FT3 and FT4 levels (Table S4). Mean FT3 and FT4 levels declined across pregnancy trimesters (Table S5). TSH levels slightly increased in women measured in the first versus the second trimester, and lowest in women measured in the third trimester.

**Table 4:** Characteristics of study participants who were pregnant with data on thyroid biomarkers in the sample and by severity levels of childhood maltreatment (n = 156).

Characteristics	Sample (n = 156)	Severity levels of childhood maltreatment			
		No (CTQ: 25 points) (n = 26)	Minimal (CTQ: 26–29 points) (n = 49)	Mild (CTQ: 30–36 points) (n = 43)	Moderate or severe (CTQ: > 36 points) (n = 38)
Age at MHMB sub-study enrollment, Mean (SD), yrs	34.4 (3.2)	34.7 (2.5)	34.0 (3.7)	34.4 (3.3)	34.8 (3.0)
Self-reported race, n (%)					
White	145 (93.0)	26 (100.0)	47 (95.9)	40 (93.0)	32 (84.2)
Other	11 (7.1)	0 (0.0)	2 (4.1)	3 (7.0)	6 (15.8)
Educational attainment, n (%)					
Bachelor or lower	58 (37.2)	11 (42.3)	16 (32.7)	11 (25.6)	20 (52.6)
Master’s or Doctorate	98 (62.8)	15 (57.7)	33 (67.4)	32 (74.4)	18 (47.4)
BMI, Median (IQR), kg/m^2^	25.2 (23.0 to 29.5)	26.0 (21.9 to 28.0)	25.0 (23.1 to 29.3)	25.1 (23.3 to 28.3)	26.2 (22.1 to 30.0)
Ever smoker, n (%)	12 (7.7)	2 (7.7)	1 (2.0)	3 (7.0)	6 (15.8)
Ever consumed marijuana, n (%)	91 (58.3)	17 (65.4)	23 (46.9)	27 (62.8)	24 (63.2)
History of cancer, n (%)	4 (2.6)	0 (0.0)	3 (6.1)	1 (2.3)	0 (0.0)
History of autoimmune disorders, n (%)	18 (11.5)	3 (11.5)	6 (12.2)	7 (16.3)	2 (5.3)
Gestational week, median (IQR)	15.6 (12.0 to 23.0)	13.0 (12.0 to 21.0)	17.5 (13.0 to 25.0)	16.0 (14.0 to 25.0)	14.0 (11.5 to 21.5)
Pregnancy trimester, n (%)					
First	39 (25.7)	11 (44.0)	10 (20.8)	7 (16.3)	11 (30.6)
Second	101 (66.5)	11 (44.0)	35 (72.9)	32 (74.4)	23 (63.9)
Third	12 (7.9)	3 (12.0)	3 (6.3)	4 (9.3)	2 (5.6)
Free T3, pmol/L, Median (IQR)	4.2 (3.8 to 4.5)	4.2 (4.0 to 4.6)	4.0 (3.7 to 4.4)	4.1 (3.6 to 4.5)	4.3 (3.9 to 4.8)
Free T4, ng/dL, Median (IQR)	1.1 (1.0 to 1.2)	1.1 (1.0 to 1.3)	1.1 (1.0 to 1.2)	1.1 (1.0 to 1.2)	1.1 (1.0 to 1.2)
TSH, uIU/mL, Median (IQR)	1.3 (0.9 to 1.8)	1.1 (0.7 to 1.4)	1.3 (1.0 to 1.9)	1.6 (1.0 to 2.1)	1.3 (0.9 to 1.7)
Positive TPOAb, n (%)					
Yes (> 9 IU/mL)	15 (9.6)	2 (7.7)	5 (10.2)	6 (14.0)	2 (5.3)
No (≤ 9 IU/mL)	141 (90.4)	24 (92.3)	44 (89.8)	37 (86.1)	36 (94.7)
Blood draw since wake up (in hours), Median (IQR)	3.8 (2.5 to 5.9)	5.0 (2.9 to 6.8)	3.8 (2.3 to 6.1)	3.3 (2.4 to 5.5)	3.4 (2.6 to 4.5)
Blood draw since last eat or drink (in hours), Median (IQR)	1.0 (1.0 to 2.0)	1.0 (1.0 to 2.0)	1.0 (1.0 to 2.0)	1.0 (1.0 to 2.0)	2.0 (1.0 to 3.0)

*MHMB* Maternal Health and Maternal Biology, *BMI* body mass index, *TSH* thyroid stimulating hormone, *IU* international unit, *TPO* thyroid peroxidase, *SD* standard deviation, *IQR* interquartile range.

#### Associations between childhood maltreatment and thyroid function

Among pregnant women, childhood maltreatment was not significantly associated with FT3, FT4 and TSH levels, after adjusting for age, gestational age, self-reported race, hours since awakening and time since last eating or drinking (Table 5, Model 1). Associations also remained non-significant after further adjusting for potential medicators (Table 5, Model 2). Additionally, childhood maltreatment was not significantly associated with positive TPOAb (Table 6). Secondarily, we did not observe significant associations between the abuse and neglect sub-scales of the CTQ and thyroid function tests in pregnant women (Table S6).

**Table 5:** Percent difference (95% CI) in FT3, FT4 and TSH levels among participants who were pregnant, according to severity levels of childhood maltreatment (n = 156).

Severity levels of childhood maltreatment	Free T3, pmol/L		Free T4, ng/dL		TSH, uIU/mL	
	Percent difference (95% CI)	p-trend^c^	Percent difference (95% CI)	p-trend^c^	Percent difference (95% CI)	p-trend^c^
Model 1^a^						
No (n = 26)	0 (Ref)	0.50	0 (Ref)	0.85	0 (Ref)	0.71
Minimal (n = 49)	− 4.1% (− 9.0% to 1.2%)		− 1.6% (− 7.2% to 4.3%)		46.2% (− 4.7% to 124.1%)	
Mild (n = 43)	− 2.5% (− 7.7% to 2.9%)		− 2.4% (− 8.1% to 3.6%)		25.3% (− 19.2% to 94.3%)	
Moderate or severe (n = 38)	0.2% (− 5.3% to 6.0%)		2.0% (− 4.1% to 8.6%)		16.3% (− 26.1% to 83.0%)	
Model 2^b^						
No (n = 26)	0 (Ref)	0.99	0 (Ref)	0.98	0 (Ref)	0.91
Minimal (n = 49)	− 3.6% (− 8.5% to 1.5%)		− 0.3% (− 6.1% to 5.9%)		48.1% (− 4.5% to 129.9%)	
Mild (n = 43)	− 3.4% (− 8.3% to 1.8%)		− 1.9% (− 7.7% to 4.2%)		23.6% (− 20.8% to 92.8%)	
Moderate or severe (n = 38)	− 0.2% (− 5.4% to 5.3%)		2.2% (− 4.0% to 8.8%)		14.0% (− 27.9% to 80.3%)	

*MHMB* Maternal Health and Maternal Biology, *CI* confidence interval, *IU* international unit.

^a^ Model 1 was adjusted for age at MHMB sub-study enrollment, race, gestational age, blood draw since wake up (in hours), and blood draw since last eat or drink (in hours).

^b^ Model 2 was additionally adjusted for educational attainment, body mass index, ever smoked, ever used marijuana, history of cancer, and history of autoimmune disorder.

^c^ p-trend was calculated by fitting models with severity of childhood maltreatment as a continuous CTQ variable.

**Table 6:** Association between severity levels of childhood maltreatment and odds of positive TPOAb levels among participants who were pregnant (n = 156).

Severity levels of childhood maltreatment	Odds of positive TPOAb (> 9 vs. ≤ 9 IU/mL)		
	Event rate, n (%)	Odds ratio (95% CI)	p-trend^c^
Model 1^a^			
No	2/26 (7.7)	1.00 (Ref)	0.46
Minimal	5/49 (10.2)	1.00 (0.17 to 5.95)	
Mild	6/43 (14.0)	1.39 (0.24 to 7.96)	
Moderate or severe	2/38 (5.3)	0.58 (0.07 to 4.69)	
Model 2^b^			
No	2/26 (7.7)	1.00 (Ref)	0.42
Minimal	5/49 (10.2)	1.39 (0.22 to 8.88)	
Mild	6/43 (14.0)	2.11 (0.34 to 13.28)	
Moderate or severe	2/38 (5.3)	0.56 (0.06 to 5.03)	

*MHMB* Maternal Health and Maternal Biology, *CI* confidence interval, *IU* international unit.

^a^ Model 1 was adjusted for age at MHMB sub-study enrollment, race, gestational age, blood draw since wake up (in hours), and blood draw since last eat or drink (in hours).

^b^ Model 2 was additionally adjusted for educational attainment, body mass index, ever used marijuana, and history of autoimmune disorder. To avoid model quasi-separation issues and undefined estimates in this model, we did not adjust for ever smoked and history of cancer.

^c^ p-trend was calculated by fitting models with severity of childhood maltreatment as a continuous CTQ variable.

## Discussion

In this observational cohort study, we examined childhood maltreatment and thyroid function in relatively healthy women of childbearing age, including both women contemplating pregnancy and those who were pregnant. Our findings indicate that among women contemplating pregnancy, childhood maltreatment was consistently associated with lower FT3 levels but was not associated with FT4, TSH, or TPOAb levels. In contrast, in pregnant women, childhood maltreatment was not associated with any thyroid function markers.

The precise molecular mechanisms through which childhood maltreatment affects long-term thyroid function are not yet fully understood. One potential mechanism is that childhood maltreatment may lead to persistent changes in the HPT axis due to stable epigenetic modifications^5,11^, which could increase the risk of thyroid dysfunction in adulthood. Another potential mechanism is that childhood maltreatment may indirectly impair thyroid function by disrupting the HPA axis. Because the HPA and HPT axes are closely linked, dysregulation in one axis may influence the other, potentially affecting thyroid hormone regulation^14^. Childhood maltreatment may also influence thyroid function through long-term effects on immune function and inflammation. Maltreatment has been associated with elevated levels of pro-inflammatory markers such as C-reactive protein, interleukin-6, and tumor necrosis factor-alpha^27–29^. These inflammatory changes can inhibit thyroid hormone synthesis and promote inflammation in thyroid tissues, which may contribute to autoimmune thyroid conditions. Based on this biological plausibility, we hypothesized that childhood maltreatment would be linked to altered thyroid function tests, including FT3, FT4, TSH, and TPOAb.

Among women contemplating pregnancy, our findings did not fully support our hypothesis. We observed lower FT3 levels in those who experienced childhood maltreatment compared to those who did not, while FT4, TSH, and TPOAb showed no associations. This specificity suggests that childhood maltreatment may influence thyroid function in a targeted way rather than causing a broad alteration. When FT3 levels are altered while FT4 levels remain unaffected, this pattern often indicates impaired peripheral conversation of FT4 to FT3 rather than a primary dysfunction of thyroid hormone production at the glandular level^30^. FT3, the biologically active and unbound form of thyroid hormone, is critical in regulating key physiological processes including metabolism, energy production, and growth^31^. Lower FT3 levels may therefore have important clinical and biological implications, potentially reflecting altered peripheral thyroid hormone metabolism, conversion of FT4 to FT3, or tissue-level thyroid hormone action^32^.

Our findings align with previous studies conducted in non-pregnant populations, including research involving adolescents and individuals with psychiatric disorders^15–18^. For example, Machado and colleagues found that among 80 adolescents, early life trauma was associated with reduced FT3 levels, but not FT4 levels^16^. Compared to previous studies^15–18^, our study included generally healthy women of childbearing age and comprehensively examined thyroid function tests to synthesis and autoimmunity and administered a 28-item CTQ incorporating five sub-scales of abuse and neglect. Separately, the non-significant association between childhood maltreatment and TPOAb positivity observed in this sample underscores the multifactorial etiology of thyroid autoimmunity. Although childhood maltreatment has been implicated in dysregulation of immune function and increased risk for various autoimmune conditions, its direct relationship with TPOAb status remains unclear^33,34^. The pathogenesis of TPOAb positivity is influenced by a complex interplay of genetic susceptibility, environmental exposures, hormonal factors, and psychosocial stressors^35,36^. It is plausible that the specific timing, severity, and type of maltreatment, as well as individual differences in stress resilience and coping mechanisms, modulate immune outcomes in ways not fully captured in this analysis^37,38^.

Despite observing lower FT3 levels in non-pregnant individuals with a history of childhood maltreatment, we found no significant associations between childhood maltreatment and thyroid function tests, including FT3, among pregnant women. The lack of association may reflect the substantial hormonal and metabolic adaptations that occur during pregnancy, which can affect maternal thyroid physiology^39^. For example, FT3 and FT4 are typically highest during the first trimester and tend to decrease slightly as pregnancy progresses, consistent with the patterns observed in our data^40^. Our findings contrast with those of Moong and colleagues (n = 102)^5^, who reported a clinically significant increase in the risk of thyroid dysfunction across pregnancy among women exposed to childhood abuse and neglect. Differences in study design may explain this discrepancy: Moong et al.^5^ measured thyroid function longitudinally across each trimester, whereas our study measured thyroid function tests at a single time point, potentially limiting our ability to detect subtle effects of childhood maltreatment on thyroid function during pregnancy.

The strengths of our study should be noted: a well-characterized sample that included both women contemplating pregnancy and those who were pregnant; measurement of thyroid function tests related to synthesis and autoimmunity; and the administration of a comprehensive 28-item CTQ assessing multiple subtypes of abuse and neglect. Our study has also limitations. First, there is the potential for recall bias and misclassification of exposure due to the retrospective assessment of childhood maltreatment. However, previous research has demonstrated high reliability in the recollection of childhood adversity experiences during adulthood^41,42^. Second, the sample consisted of relatively healthy women of childbearing age with limited racial diversity. As a result, the findings may not be generalizable to women from other sociodemographic and clinical groups, potentially affecting the generalizability of our findings. Additionally, most participants had thyroid function test results within the normal laboratory reference range, which limits our ability to assess the relationship between childhood maltreatment and clinically relevant thyroid conditions (e.g., hypothyroidism). Third, thyroid function test values can fluctuate depending on the phase of the menstrual cycle when blood is collected^43,44^. In the subset of women contemplating pregnancy, we did not measure thyroid function tests during specific phases, such as the follicular or luteal phases. This could affect the interpretation of our findings. Similarly, although ~ 70% of samples from pregnant participants were obtained during the second trimester, variation in gestational age at blood draw may have introduced some variability in thyroid hormone levels. Since thyroid function reference ranges vary throughout pregnancy, this limits the precision of interpreting thyroid measures and may have attenuated potential associations with childhood maltreatment in this subgroup. Fourth, we did not collect information on childhood socioeconomic status or parity as a potential confounder in this study, which might limit our ability to fully understand the influence of these factors on the relationship between childhood maltreatment and thyroid function^45^.

In conclusion, among women contemplating pregnancy, childhood maltreatment was associated with lower FT3 levels, but not FT4, TSH or TPOAb levels. However, in pregnant women, we found no significant differences in thyroid function test values in relation to childhood maltreatment. These findings are observational in nature, and the underlying biological mechanisms remain to be elucidated. Future large, prospective studies including more racially and ethnically diverse or socioeconomically disadvantaged individuals are needed to confirm our findings.

## Supplementary Information

Supplementary Information.

## Data Availability

Access to de-identified data, data dictionaries to code used to generate study data will be granted within the study’s data repository system after obtaining authorization from our local IRB and putting in place Data Use Agreements with the parties interested in accessing study data Further information including the procedures to obtain and access data from the Nurses’ Health Studies is described at https://www.nurseshealthstudy.org/researchers (contact email: nhsaccess@channing.harvard.edu).
