# Supplementary material for: Associations between women’s childhood maltreatment and thyroid function before and during pregnancy
Source: Sci Rep. 2026 Apr 25;16:19116. doi: 10.1038/s41598-026-48820-9 (PMC13279924; doi:10.1038/s41598-026-48820-9)
Supplement: Supplementary file 1 — Supplementary Information. [file 41598_2026_48820_MOESM1_ESM.docx]

**Associations between women’s childhood maltreatment and thyroid function before and during pregnancy**

**Vyas CM et al.**

**Online Supplement**

**Table S1. Spearman-rank correlations between thyroid function tests among women contemplating pregnancy.**

**Table S2. Spearman-rank correlations between age and thyroid function tests among women contemplating pregnancy.**

**Table S3. Associations between individual CTQ sub-scales with thyroid function tests among women contemplating pregnancy (n=219).**

**Table S4. Spearman-rank correlations between thyroid function tests among pregnant women.**

**Table S5. Mean (range) thyroid function test levels according to pregnancy trimester.**

**Table S6. Associations between individual CTQ sub-scales with thyroid function tests among pregnant women (n=156).**

**Table S1. Spearman-rank correlations between thyroid function tests among women contemplating pregnancy.**

| Thyroid function test |  | FT3, pmol/L | FT4, ng/dL |
| --- | --- | --- | --- |
| FT3, pmol/L | Rho | - |  |
|  | p-value |  |  |
| FT4, ng/dL | Rho | 0.39 | - |
|  | p-value | <0.0001 |  |
| TSH, uIU/mL | Rho | -0.02 | -0.13 |
|  | p-value | 0.79 | 0.05 |

Abbreviation: FT3, free triiodothyronine; TSH, thyroid-stimulating hormone; FT4, free thyroxine

**Table S2. Spearman-rank correlations between age and thyroid function tests among women contemplating pregnancy.**

|  |  | FT3, pmol/L | FT4, ng/dL | TSH, uIU/mL |
| --- | --- | --- | --- | --- |
| Age, yrs | Rho | -0.2 | -0.12 | 0.04 |
|  | p-value | 0.003 | 0.07 | 0.6 |

Abbreviation: FT3, free triiodothyronine; TSH, thyroid-stimulating hormone; FT4, free thyroxine

**Table S3. Associations between individual CTQ sub-scales with thyroid function tests among women contemplating pregnancy (n=219).**

**A) FT3, FT4, TSH**

| Individual CTQ subscale (continuous score, in points) | Free T3, pmol/L | | Free T4, ng/dL | | TSH, uIU/mL | |
| --- | --- | --- | --- | --- | --- | --- |
|  | Percent difference (95% CI)^a^ | p-value | Percent difference (95% CI)^a^ | p-value | Percent difference (95% CI) ^a^ | p-value |
| Emotional abuse | -0.2% (-0.5% to 0.2%) | 0.43 | 0.1% (-0.3% to 0.5%) | 0.64 | -0.4% (-1.8% to 1.1%) | 0.62 |
| Physical abuse | 0.0% (-0.9% to 0.9%) | 0.97 | 0.0% (-1.0% to 1.0%) | 0.99 | -0.2% (-3.5% to 3.3%) | 0.93 |
| Sexual abuse | 0.1% (-0.5% to 0.6%) | 0.85 | 0.0% (-0.7% to 0.6%) | 0.92 | 0.0% (-2.2% to 2.3%) | 0.99 |
| Emotional neglect | -0.3% (-0.7% to 0.1%) | 0.13 | 0.2% (-0.2% to 0.7%) | 0.26 | 0.1% (-1.3% to 1.6%) | 0.86 |
| Physical neglect | -0.2% (-0.9% to 0.6%) | 0.64 | 0.5% (-0.3% to 1.3%) | 0.26 | -0.1% (-2.9% to 2.9%) | 0.96 |

**B) TPOAb**

| Individual CTQ subscale (continuous score, in points) | Odds of reporting positive TPOAb (>9 vs. ≤9 IU/mL) | | |
| --- | --- | --- | --- |
|  | Odds ratio (95% CI)^a^ | p-value |  |
| Emotional abuse | 1.01 (0.90 to 1.14) | 0.88 |  |
| Physical abuse | 0.95 (0.68 to 1.31) | 0.74 |  |
| Sexual abuse | 0.89 (0.65 to 1.21) | 0.45 |  |
| Emotional neglect | 0.98 (0.86 to 1.11) | 0.74 |  |
| Physical neglect | 0.72 (0.43 to 1.22) | 0.22 |  |

^a^ Model was adjusted for age at MHMB study enrollment, race, blood draw since wake up (in hours), and blood draw since last eat or drink (in hours).

**Table S4. Spearman-rank correlations between thyroid function tests among pregnant women.**

| Thyroid function test |  | FT3, pmol/L | FT4, ng/dL |
| --- | --- | --- | --- |
| FT3, pmol/L | Rho | - |  |
|  | p-value |  |  |
| FT4, ng/dL | Rho | 0.50 | - |
|  | p-value | <0.0001 |  |
| TSH, uIU/mL | Rho | -0.08 | -0.13 |
|  | p-value | 0.31 | 0.11 |

Abbreviation: FT3, free triiodothyronine; TSH, thyroid-stimulating hormone; FT4, free thyroxine

**Table S5. Mean (range) thyroid function test levels according to pregnancy trimester.**

| Thyroid function test | Mean (min – max) | | |
| --- | --- | --- | --- |
|  | First trimester (n=39) | Second trimester (n=101) | Third trimester (n=12) |
| Free T3, pmol/L | 4.40 (3.56 to 5.37) | 4.15 (3.07 to 5.95) | 3.72 (3.31 to 4.46) |
| Free T4, ng/dL | 1.16 (0.94 to 1.53) | 1.08 (0.76 to 1.44) | 0.94 (0.73 to 1.06) |
| TSH, uIU/mL | 1.44 (0.13 to 4.80) | 1.45 (0.004 to 4.40) | 1.23 (0.40 to 3.01) |

Abbreviation: FT3, free triiodothyronine; TSH, thyroid-stimulating hormone; FT4, free thyroxine

**Table S6. Associations between individual CTQ sub-scales with thyroid function tests among pregnant women (n=156).**

**A) FT3, FT4, TSH**

| Individual CTQ subscale (continuous score, in points) | Free T3, pmol/L | | Free T4, ng/dL | | TSH, uIU/mL | |
| --- | --- | --- | --- | --- | --- | --- |
|  | Percent difference (95% CI)^a^ | p-value | Percent difference (95% CI)^a^ | p-value | Percent difference (95% CI) ^a^ | p-value |
| Emotional abuse | 0.0% (-0.5% to 0.5%) | 0.97 | 0.1% (-0.5% to 0.6%) | 0.82 | 1.0% (-2.9% to 5.0%) | 0.63 |
| Physical abuse | 0.4% (-0.9% to 1.6%) | 0.58 | -0.6% (-1.9% to 0.8%) | 0.43 | -1.1% (-10.5% to 9.3%) | 0.83 |
| Sexual abuse | 0.2% (-0.4% to 0.9%) | 0.45 | 0.0% (-0.7% to 0.7%) | 0.94 | 1.5% (-3.5% to 6.8%) | 0.56 |
| Emotional neglect | 0.3% (-0.2% to 0.8%) | 0.28 | 0.4% (-0.2% to 0.9%) | 0.20 | 0.4% (-3.7% to 4.7%) | 0.84 |
| Physical neglect | 0.0% (-0.9% to 0.9%) | 0.98 | -0.5% (-1.5% to 0.5%) | 0.31 | -0.1% (-7.1% to 7.4%) | 0.97 |

**B) TPOAb**

| Individual CTQ subscale (continuous score, in points) | Odds of reporting positive TPOAb (>9 vs. ≤9 IU/mL) | | |
| --- | --- | --- | --- |
|  | Odds ratio (95% CI)^a^ | p-value |  |
| Emotional abuse | 0.96 (0.81 to 1.14) | 0.65 |  |
| Physical abuse | 0.75 (0.43 to 1.30) | 0.30 |  |
| Sexual abuse | 0.95 (0.74 to 1.23) | 0.71 |  |
| Emotional neglect | 0.98 (0.83 to 1.17) | 0.85 |  |
| Physical neglect | 0.67 (0.35 to 1.30) | 0.24 |  |

^a^ Model was adjusted for age at MHMB study enrollment, gestational age, race, blood draw since wake up (in hours), and blood draw since last eat or drink (in hours).
